# Adolescent dysmenorrhoea in general practice: tensions and uncertainties

**DOI:** 10.3389/frph.2024.1418269

**Published:** 2024-08-23

**Authors:** Sharon Dixon, Neda Taghinejadi, Claire Duddy, Flora Holloway, Katy Vincent, Sue Ziebland

**Affiliations:** ^1^ Nuffield Department of Primary Care Health Sciences, University of Oxford, Oxford, United Kingdom; ^2^ Nuffield Department of Women's Reproductive Health, Oxford, United Kingdom; ^3^ School of Social and Political Sciences, University of York, York, United Kingdom

**Keywords:** adolescent & youth, dysmenorrhoea, period pain, medical sociology, sociology of diagnosis, general practice

## Abstract

This Perspectives article reflects on findings from our systematic review about adolescent dysmenorrhoea Q, drawing on sociology of diagnosis theory. We consider tensions and uncertainties between presentation with symptoms of dysmenorrhoea and processes of symptom categorisation and diagnosis in adolescents, tracing these through research and clinical guidance, considering possible implications for clinical practice. We argue that challenges in distinguishing between primary and secondary dysmenorrhoea in research translate into challenges in differentiation in clinical practice. We argue that framing this distinction as clear cut and straightforward belies the well-documented challenges in diagnosis of endometriosis, and that not recognising uncertainty and complexity inherent in this task may benefit neither clinicians nor patients.

## Introduction

The worldwide prevalence of menstrual pain (dysmenorrhoea) amongst teenagers who menstruate is strikingly high; up to 94% (1–3) report menstrual pain, with one third reporting severe pain (4). More than 20.1% of menstruating adolescents regularly miss school because of dysmenorrhoea (2, 5), and when able to be present at school menstrual pain reduces young people's ability to study and concentrate (1).

Dysmenorrhoea can occur without an identified physical cause (primary dysmenorrhoea) or be associated with an underlying condition (secondary dysmenorrhoea). In adolescence, conditions causing secondary dysmenorrhoea include endometriosis, developmental genital tract anomalies, and pelvic inflammatory disease (6, 7). Once thought rare in adolescence, endometriosis is an important (8) cause of secondary dysmenorrhoea in this age group (9, 10). The community prevalence of adolescent endometriosis is unknown (11), with most evidence from tertiary or specialist referral centres (10, 12–14). Adults and adolescents alike experience well-documented delays between presentation with symptoms and a diagnosis of endometriosis (9). The interface between menstrual pain and possible endometriosis, especially for adolescents, has been identified as an area of clinical uncertainty for GPs (15).

Distinguishing between primary and secondary dysmenorrhoea is a pivotal task when assessing dysmenorrhoea, often depicted as a (relatively) straightforward dichotomous bifurcation, to be made at the outset of assessment, on the basis of proposed characteristics that delineate primary from secondary dysmenorrhoea (7).

In this Perspectives article, we draw on our systematic review (16) and sociological writing about diagnosis (17), to reflect on inconsistencies in evidence relevant to the differentiation between primary and secondary dysmenorrhoea, and consider potential implications for care.

Diagnosis is “the making of a judgment about the exact character of a disease or other problem, especially after an examination, or such a judgment”. The term diagnosis encompasses both the process and the endpoint of assessment. Diagnoses function as classification tools which organise health conditions, categorised by a range of features (symptom, site, cause, variation from statistical norm) (18). Symptoms, by contrast, are subjectively experienced bodily sensations with potential to be relevant for a diagnosis within the context of accessing healthcare (19).

Diagnoses, both classification tools and process, are not static but dynamic and respond to evolutions of knowledge, understanding, and process (18). They contribute to delineating what is deemed normal (acceptable), and what is abnormal (pathological), and as such are embedded and responsive to temporal and social contexts. Historical examples illustrating diagnoses at the interface between scientific knowledge (objective, dispassionate) and cultural context include homosexuality and hysteria, which were previously considered diseases (17). These examples also illustrate the power implicit within the giving and naming of diagnoses, power which resides in bodies of authority such as medical profession, the state, and religion.

Consequences of a diagnosis can include medical impacts, for example which professionals become involved in care and which treatments are offered, but also wide-ranging social and legislative sequelae, including insurance cover, permission to become a patient, experience validation and membership of communities connected by shared diagnoses (18).

Within a framework positioning diagnoses as pivot-points in medicine, is the implicit idea that diagnoses makes sense of symptoms, elevating them from subjectively experienced sensations to something defined and objective, but also abnormal, or pathological. Diagnoses become tickets which transition illness (experience) to disease (objective medical entity). Diagnoses are situated at the conclusion of clinical reasoning, but diagnoses are not the endpoint of a journey through healthcare, but are also a starting point, serving as a gateway for evidence-based treatment, enrolment in clinical trials, or membership of clinical communities and support groups.

## The diagnosis of dysmenorrhoea

The framing of dysmenorrhoea as symptom or diagnosis has evolved in response to scientific knowledge and societal contexts. The conceptualisation of “normal” menstruation in gynaecology was initially constructed from clinical encounters with women who identified themselves as having an abnormal or problematic menstruation. Menstrual pain was considered near universal, and the inevitability of menstrual discomfort was utilised to support arguments against female education because women were “more or less sick and unfit for hard work” for one quarter of each month (20). Language equating menstruation with a time of illness exemplifies this and persists today.

With increasing attention to women's rights, menstruation was re-framed as a natural function, not an illness. Women were advised that menstruation was “not normally accompanied by pain or malaise” and that they should carry on with education and work, alongside messages advising discretion and concealment, a menstrual etiquette that persists (20, 21).

During the 1960's and 1970's, adolescent dysmenorrhoea was typified in western medical writing as predominantly psychosomatic: a maladaptive response to normal menstrual function or adolescence, rather than a medical condition (22–25). Some related dysmenorrhoea to developing or rejecting femininity (25, 26). In this worldview, teenagers needed to be supported with menstrual pain until mature enough to embrace their femininity or womanhood (22, 23, 27, 28), a process which could reportedly be made problematic through modelling of the sick role by over-protective mothers (24, 28–30). Differences in experience of dysmenorrhoea by social class were related to this, for example positioning dysmenorrhoea as a privileged indulgence, not seen in “primitive” societies (22, 29).

As knowledge evolved, showing that dysmenorrhoea was *caused* by chemicals/transmitters (prostaglandins and leukotrienes) acting on menstrual endometrium and muscle motility (8, 31–35), these theories lost dominance, and dysmenorrhoea transitioned from being considered a psychosocial to a medical issue (23, 24, 36, 37). Zola writes that once a diagnosis relates to bodily malfunction (for example excess prostaglandins in dysmenorrhoea), then it comes within medical jurisdiction (38), illustrated here as dysmenorrhoea transitions from psychodynamic construct towards medical entity.

Within medicine, the International Classification of Disease (ICD) seeks to make the classification of disease consistent and objective (39). Dysmenorrhoea is defined within the category “*diseases* of the genitourinary tract”, summarised in Box 1 (40).

Box 1 **ICD classification and positioning of dysmenorrhoea.**

Dysmenorrhoea is defined within this family tree: *Diseases of the genitourinary system*

*GA34 Female pelvic pain associated with genital organs or menstrual cycle*

*GA34.3 Dysmenorrhoea*

And is defined as follows within the ICD-11:

*A condition of the genital system affecting females, caused by endometriosis, adenomyosis, ovarian cysts, or may be idiopathic. This condition is characterised by cyclic pelvic pain preceding or accompanying menstruation that interferes with daily activities, lower, umbilical, or suprapubic abdominal pain, such as sharp, throbbing, burning, or shooting pains that may extend to the thighs and lower back* ^1^

Here, dysmenorrhoea is arguably both diagnosis and symptom: a *genital tract condition* characterised by *symptom(s)*, which become pathological once they have *functional impacts* on daily activities and which may be related to anatomy [*an identifiable pathological cause or can be idiopathic* (no identifiable cause)]. Thus “diagnosis” of dysmenorrhoea is simultaneously categorised or defined by body site, symptom experience, symptom impact and structural (pathological) cause (or not). Primary and secondary dysmenorrhoea are not differentiated in this definition, but aligned under a symptomatic heading, with separate ICD-11 definitions for causes of secondary dysmenorrhoea (40). This unified definition of dysmenorrhoea explicitly affords medical validity to all menstrual pain, whatever the cause (and where no cause is identified), worthy of (medical) recognition and treatment.

However, having “diagnosed” dysmenorrhoea, the subsequent clinical task of differentiating primary from secondary dysmenorrhoea remains pivotal in medical discourse and guidance. The American College of Obstetricians and Gynaecologists flowchart for “managing the adolescent with dysmenorrhoea” begins with the bifurcation; “history suggests primary dysmenorrhoea” vs. “symptoms or history suggestive of secondary dysmenorrhoea” (7). NICE Clinical Knowledge Summaries begin their guidance saying: “secondary causes of dysmenorrhoea must be excluded before considering a diagnosis of primary dysmenorrhoea” (41). Note the language; primary dysmenorrhoea as diagnosis, not symptom, implying conclusion about the (lack of identifiable) cause for the symptom.

This differentiation is frequently recounted within the introduction to papers on adolescent dysmenorrhoea, as a pivotal distinction, essential for both scientific exploration and clinical care (6, 8, 34, 42), although there is recognition that secondary dysmenorrhoea can be associated with the same clinical features, albeit associated with pelvic pathology (43).

However, while the core definitions of primary and secondary dysmenorrhoea are largely consistent, we identify variability in how these diagnoses are accounted for and further characterised in research inclusion and recruitment. While some clinical associations that could assist in differentiating primary from secondary dysmenorrhoea, such as congenital anomalies of the urogenital tract, were consistently identified as helpful, we identified inconsistencies in others (16), often cited with relative certainty (6, 8, 31, 43, 44–47). These tensions and inconsistencies are listed in Box 2, and then further detailed below:^1^

Box 2 **Areas of tension and uncertainty relevant to the differentiation of primary and secondary dysmenorrhoea.**

• Inclusion criterion for studies documenting evidence about primary dysmenorrhoea
• The relationship between prostaglandins and menstrual pain
• The relationship between menarche and onset of menstrual pain
• The association between regular (ovulatory) cycles and menstrual pain
• Response to trials of treatment in diagnostic pathways.
• Acyclic pelvic pain
• Normal or not normal menstrual pain (healthy or un-healthy adolescents)?

## Areas of tension and uncertainty relevant to the differentiation of primary and secondary dysmenorrhoea

### Inclusion criterion for studies documenting evidence about primary dysmenorrhoea

We identified considerable incongruence in how authors defined study populations, and reported empirical evidence about primary dysmenorrhoea (16). While some differences reflect specific research aims, the net result is a body of evidence in which primary dysmenorrhoea is defined and operationalised inconsistently. These are described in detail in our systematic review, but we note that of 73 papers reporting evidence about primary dysmenorrhoea; 37/73 relied on participant self-report, 9/73 offered no detail on the differentiation between primary and secondary dysmenorrhoea 2/73 assumed primary dysmenorrhoea based on (young) age, 6/73 used health professional assessment without imaging, 12/73 used ultrasound and/or biomarkers, and 6/73 were systematic reviews. We identified one paper (representing 1/40,390 participants) which reported a negative laparoscopy (16).

The vast majority of these approaches would not reliably identify all secondary dysmenorrhoea, raising the possibility that studies characterising primary dysmenorrhoea are actually reporting undifferentiated dysmenorrhoea (16).

### The relationship between prostaglandins and menstrual pain

The causal role of prostaglandins is frequently embedded in descriptions of primary dysmenorrhoea (16), often contrasted with secondary dysmenorrhoea, where pain is attributed to the underlying condition (7, 48–50). However, prostaglandins and inflammatory cytokines likely contribute to the uterine contractility and pain associated with both primary and secondary dysmenorrhoea including endometriosis-associated pain (9, 47, 51, 52). Medications that act on the prostaglandin pathway (NSAIDs) or reduce menstruation associated release of prostaglandins, among other actions (hormonal contraceptives) are recommended treatments for both primary dysmenorrhoea and secondary dysmenorrhoea including endometriosis (6, 7, 44, 53–55), although there is less certainty about trial evidence supporting the efficacy of NSAIDs in endometriosis-associated pain (56).

### The relationship between menarche and onset of menstrual pain

We identified a wide range of assertions about the timing between onset of menstrual pain after menarche and the likelihood of the pain being primary or secondary, including that (primary dysmenorrhoea) menstrual pain is expected to onset within 6–12 months, after 6–12 months, within 6–24 months, within 12–36 months or after 12–36 months (or several years) (16).

Some suggest that onset of pain from menarche is abnormal (7, 34, 43, 57) and should be investigated, however in some empirical studies, a significant proportion of adolescents reported pain starting from menarche (58–61). Secondary dysmenorrhoea can be depicted as rare in adolescence (62), with onset several years after menarche (63), whilst others advise that pain starting after 2 years from menarche is most likely to be secondary and the cause should be “vigorously sought” (6). Even with congenital anomalies of the uterus, where pain is likely to onset with menarche (16, 43), our review of case reports demonstrate variable onset of this type of secondary dysmenorrhoea, including several years after menarche (16).

However, it is also noteworthy that a cross sectional study with adolescents with endometriosis did find that 50% of adolescents with endometriosis had menstrual pain from menarche (64).

### The association between regular (ovulatory) cycles and menstrual pain

It is often reported that primary dysmenorrhoea commences when regular (ovulatory) cycles become established, at some point after menarche, casting ovulatory cycles as a causative mechanism for primary dysmenorrhoea (16). Recent studies cast doubt on this relationship, with evidence that many non-ovulatory cycles are painful (65) and that non-ovulatory cycles are potentially both painful and common in adolescents (66).

### Response to trials of treatment

Response (or not) to empirical treatment is another tool proposed to help discriminate between primary dysmenorrhoea and secondary dysmenorrhoea, where response to treatment aligns with primary dysmenorrhoea and therefore suggesting that lack of response to empirical therapy said to increase the likelihood of pathology (7, 44, 67). Trials of treatment with anti-prostaglandin medication (NSAIDS) or menstrual suppression with hormonal contraceptive therapy are embedded in national and international guidance on adolescent endometriosis; suggesting onward referral for specialist investigation is these are not successful in relieving symptoms (7, 53, 54). This raises the question about what clinicians and patients should do if these treatments *are* effective in alleviating dysmenorrhoea (15).

The first-line medications (NSAIDs, hormonal contraception) recommended for dysmenorrhoea and endometriosis are the same, creating a paradox: when symptoms are effectively treated, the grounds for referral for diagnosis are removed, yet an evidence-based treatment for the condition once diagnosed is the same as that trialled empirically for symptomatic relief without a diagnosis.

However, the evidence associating non-response to hormonal contraception therapy as a marker of likely endometriosis is inconsistent. In retrospective case series, where the cases have a surgically confirmed diagnosis of endometriosis, many had dysmenorrhoea or pelvic pain which was refractory to medical treatment (13, 68). But, two prospective case series following young people with marked dysmenorrhoea suggest that many achieve symptomatic responses to hormonal therapy, including 92% achieving a positive symptomatic response to empirical treatment in one specialist clinic (50, 69).

Strikingly, in one case series, the likelihood of endometriosis in adulthood positively correlated with a positive therapeutic response to hormonal treatment for dysmenorrhoea in adolescence, i.e., having a good response to empirical treatment was a marker for having endometriosis, not non-response as currently embodied in guidance (69). A other prospective study we identified excluded all adolescents with a positive response to therapy from further investigations (70), thus arguably perpetuating the construct that this equates to a lack of pathology.

While not specific to adolescents, a systematic review, and meta-analysis, found that current use of CHC reduced the likelihood of an endometriosis diagnosis, whilst past or ever use increased the risk (71). Aligned with this, in a case series of 410 women the risk of being diagnosed with deep infiltrating endometriosis in adulthood was significantly higher (OR 5.6) for those treated with hormonal contraception for “primary dysmenorrhoea” in adolescence (72). Both recognised that this may in part be because CHC use may reduce symptoms of dysmenorrhoea and defer diagnostic processes or because these prescriptions are acting as a marker for the presence of intractable symptoms.

The temporality and use of the word primary here is noteworthy. Dysmenorrhoea, including severe dysmenorrhoea in adolescence, is statistically a risk factor for endometriosis (73), but as with this example, there is uncertainty as to whether it was a risk factor contributing to the later development of endometriosis, or an earlier symptom suggesting that endometriosis was already present. This is a known unknown (73), but we reflect that this uncertainty may be masked by use of the term primary. There are examples of linguistic positionality, where “primary dysmenorrhoea” (not dysmenorrhoea) is situated as a possible precursor of endometriosis (74), or where secondary cases become apparent by increasing dysmenorrhoea or that describes endometriosis as an important cause of both primary and secondary dysmenorrhoea (75).

### Acyclic pelvic pain

Acyclic pelvic pain is an important predictor of secondary dysmenorrhoea. Chronic or treatment resistant pelvic pain is shown to be highly predictive of endometriosis (64, 68), and as such, is a symptom that should trigger referral. But, when looking at prospective evidence from community surveys, 19%–30% of teenagers report pelvic pain outside of menstruation (4, 76, 77).

### Normal or not normal menstrual pain (healthy or un-healthy adolescents)?

Studies exploring adolescent dysmenorrhoea sometimes contrast cases (those with reported, documented or declared dysmenorrhoea) with “healthy” or normal controls (those without dysmenorrhoea) (66, 78, 79). This arguably positions adolescents with dysmenorrhoea as *un*healthy, possessed of disease, and deviants from normal. The term normal describes symptoms or sensations expected as part of “healthy” or non-pathological menstruation. Working out when menstrual pain is normal or not is embedded in education and guidance for young people about when they should seek care (80), but this can be difficult to interpret, including for health professionals (81). One of the authors, an adolescent lived experience project adviser, explored the internet using their own searches to try to find information about whether to seek help for period pain. Their experience is summarised in Box 3.

Box 3 **Working out whether pain is (might be) normal (or not); a young person's perspective.**

How: I Googled ‘what makes a period not normal', ‘period pain', ‘is my period pain normal', ‘when should I see a Dr about period pain', ‘is my period normal?', ‘Do I have normal period pain?', ‘Why is my period pain so bad?’ I looked at what came up first as information and resources, and I made notes on the first page of hits (approximately ten per question).

Observations:

• I noticed that advice on whether pain was normal was primarily about possible impacts of pain and functioning. I found this hard to make sense of and sometimes there was not much more detail given (I wasn't sure what was meant by coping or not being able to go about my day, for example was this with or without painkillers).
• Often websites would have messages both claiming that period pain is normal and that I should see my GP if my pain was not normal. Although there was sometimes a threshold for this (for example severity, sudden increases in pain, pain not better after three months of medication), I found this potentially difficult to navigate.
• I noticed that resources for education settings tended to position period pain as normal, and focus on biology rather than the impacts pain may have on life.
• I observed that advice for younger people often advised them to talk to parents or teachers.

## Discussion

These inconsistencies in evidence and terminology represent uncertainties about the symptoms that make a diagnosis of primary or secondary dysmenorrhoea more likely. When these depictions become embedded in clinical guidance and risk prediction tools, they risk entrenching underpinning assumptions.

**Figure 1:**
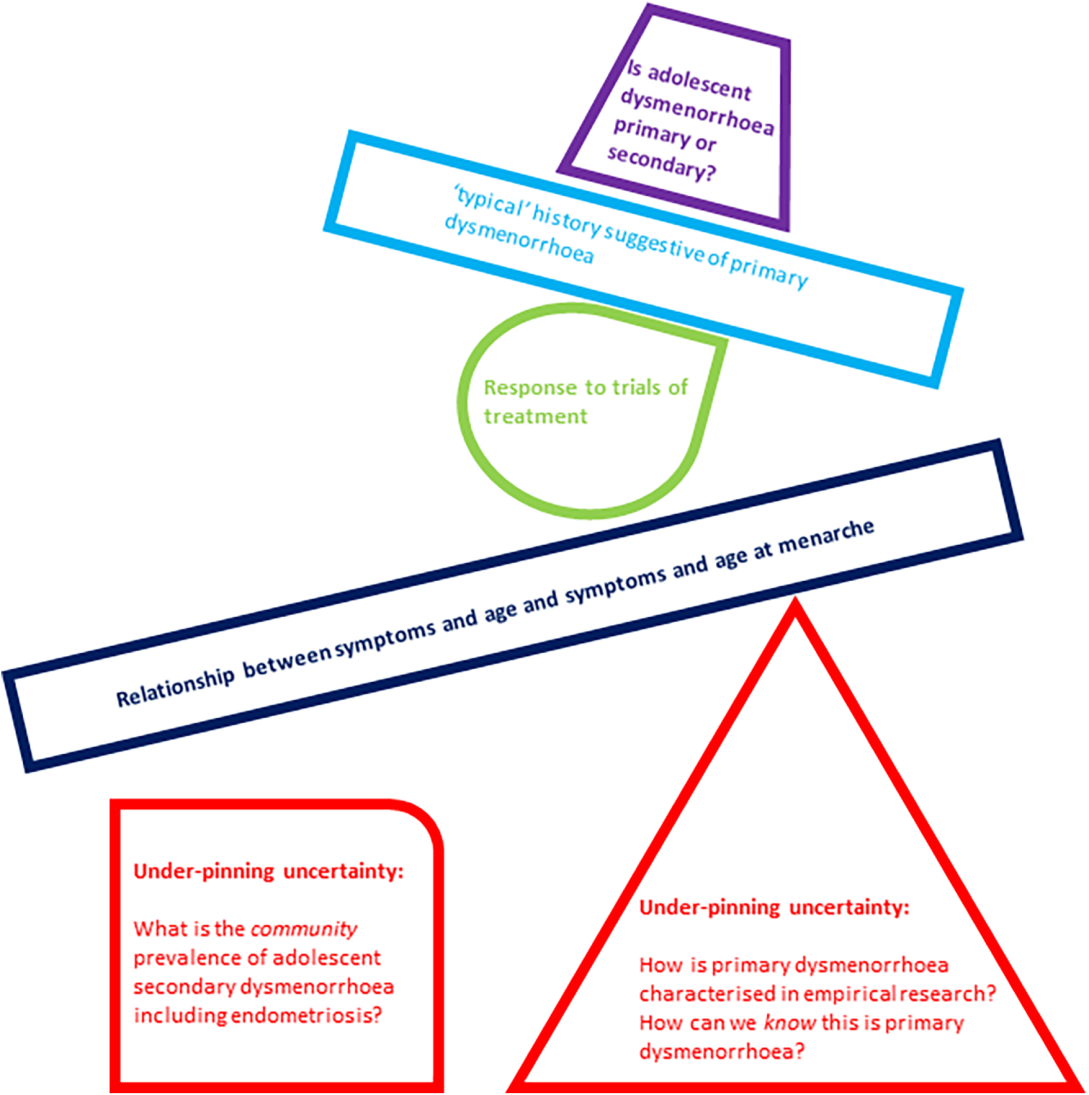
Graphical representation of under-pinning uncertainties in differentiating primary from secondary dysmenorrhoea creating an unstable base for subsequent suggested clinical tools and building blocks in this task.

For clinicians negotiating the interface between menstrual pain and possible endometriosis, we think this highlights a real-world challenge. The diagnostic criterion for primary dysmenorrhoea arise from studies which likely include cases of both primary and secondary dysmenorrhoea. Potential triggers for investigation for endometriosis are identified from the retrospective accounts of those with known endometriosis. We do not know enough about the community prevalence of endometriosis or about the symptoms or experience of adolescents without diagnosed endometriosis. This results in a diagnostic framework built upon an unstable foundation, and is depicted figuratively in Figure 1.

Representations of dysmenorrhoea have shifted from pain being near universal to relatively rare back to common (normal), been co-opted within wider social and political domains, and embedded within cultural representation and expectations along gendered lines (20, 82, 83). Menstrual etiquette requiring discretion and concealment persists, and is implicated in delays in diagnosis for endometriosis (84).

Normalising pain is proposed as an explanation for why young people don't ask for support with dysmenorrhoea (85), and why doctors don't offer it. We consider this a question not an answer; we need to understand what maintains and creates normalisation of dysmenorrhoea across settings to learn how to dismantle this and effectively enable care.

Reflections on when menstrual pain is normal or abnormal sit alongside medical discourse about the diagnosis of primary and secondary dysmenorrhoea. A critical uncertainty is how, when, and whether depictions of abnormal menstrual pain predict secondary dysmenorrhoea, and whether normal menstrual pain predicts primary dysmenorrhoea. Young people may consider their menstruation normal, even when clinicians do not and vice versa. Does considering pain normal impact on care seeking and health encounters? Does a clinician diagnosing pain as primary dysmenorrhoea risk closing down clinical curiosity and dialogue? Does this risk creating a hierarchy that pain which is (or might be) associated with identifiable pathology becomes more likely to be treated or seen as a valid reason for seeking care.

If the focus of care becomes diagnostic, rather than symptom-focussed, do we risk an unintended consequence of deferring care seeking for pain considered normal?

**Figure 2:**
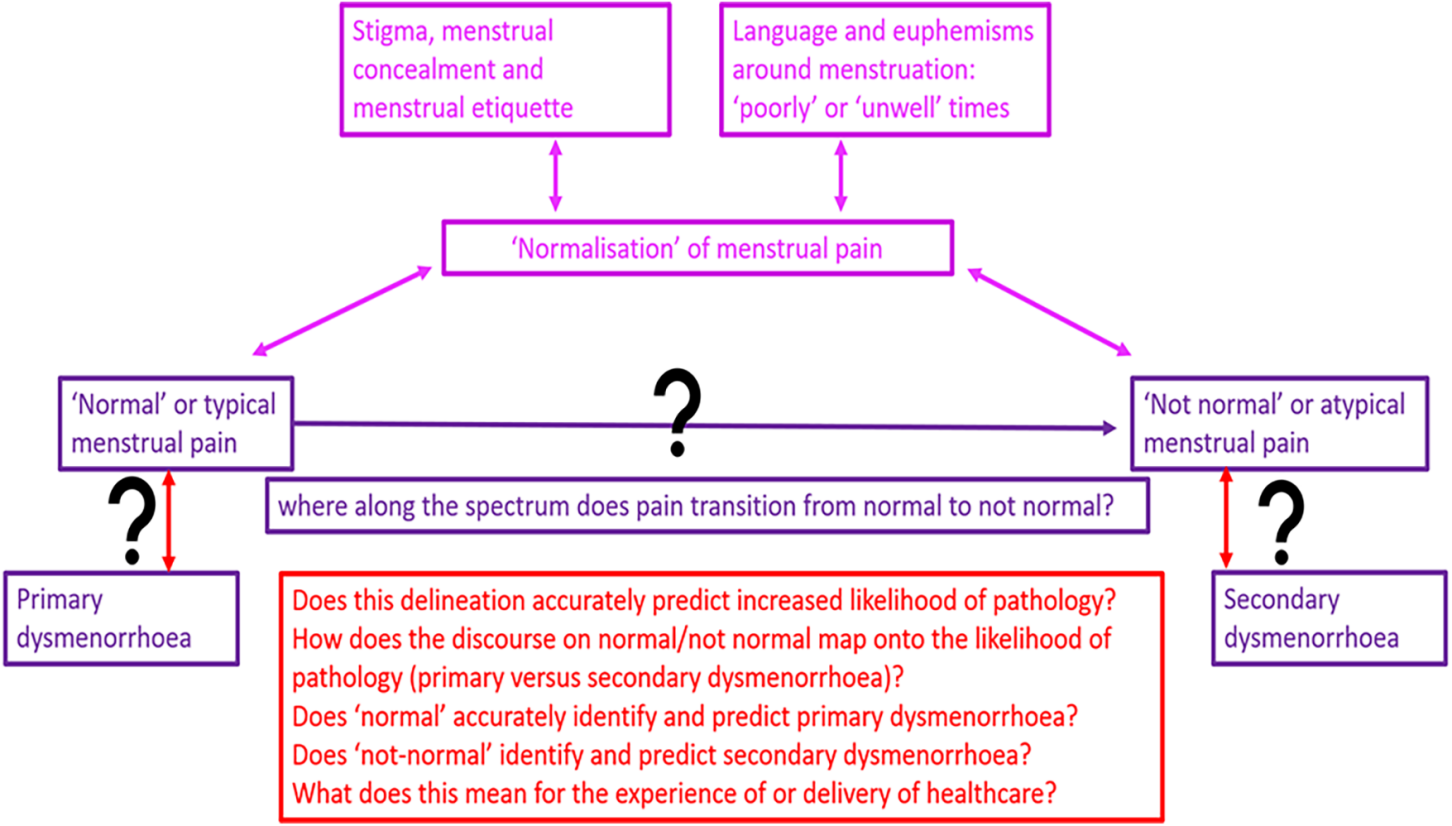
Schematic representations of questions, tensions and uncertainties in differentiating primary from secondary dysmenorrhoea.

These questions and tensions are summarised schematically in Figure 2.

## Conclusion

While the term “primary dysmenorrhoea” functions as a diagnostic label, predicated upon a belief about the (lack of) an underlying physical cause, the scientific framework that elevates it from the undifferentiated symptom (dysmenorrhoea) is imperfect and incomplete. This translates into challenges in clinical practice.

Words and semantics matter, especially if they imply (to clinicians, young people and their families, and society) that the cause of the pain is known, whereas in reality there is only a working hypothesis which might change. This risks constraining clinical curiosity and diagnostic reasoning and may impact on research, how clinical trials of treatment are negotiated and experienced and lose sight of the aim to treat all women with pain.

A strength of this perspectives article is that it brings together clinicians, (including from gynaecology, sexual health and general practice), a social science expert, and a lived experience adviser to reflect critically on findings from our systematic review. We utilise insights from clinical practice and sociological theory to illustrate possible impacts for young people accessing services, where clinical and research findings intersect with service experience.

Possible implications span research and clinical practice. There is increasing attention paid to the potential challenges in identifying primary dysmenorrhoea in research, as evidenced by recent Cochrane systematic reviews reporting evidence about primary dysmenorrhoea. These cite uncertainty about inclusion and potential heterogeneity of participants with primary dysmenorrhoea as a potential limitation, but this is not universal (86, 87). While the terms primary and secondary dysmenorrhoea are retained in academic and clinical discourse, we consider that guidance, both in standards for defining and reporting on primary dysmenorrhoea in research inclusion, could be valuable and would help both researchers, and clinicians who utilise this evidence in their work. Alternatively, we argue that moving towards using the term undifferentiated dysmenorrhoea may more closely reflect clinical reality. This includes uncertainty about who may have underlying endometriosis, which teenagers should or could be further investigated and when, including when their presenting symptoms are adequately managed by empirical or first line medical therapy.

This is a known unknown, but would be critically helpful in guiding care and shared-decision making with young people. We need prospective studies that document the natural history of adolescent dysmenorrhoea, beginning with symptoms, to add nuance to what we understand from retrospective evidence from those with diagnosed endometriosis. This will support the development of a more precise understanding of who may have primary or secondary dysmenorrhoea and how this can be predicted and mapped onto guidance. Clinical trials that evaluate the impact of hormonal therapy on long-term outcomes when symptoms are controlled are also sorely needed.

This knowledge gathering could be facilitated by the development of new options for testing and diagnosis of endometriosis, including non-surgical and non-invasive tests. These innovations could enable re-definition of the landscape of understanding about adolescent dysmenorrhoea, including when it may indicate possible endometriosis, which would be welcome. This would however need to be accompanied by a re-evaluation of guidance to ensure that it reflects reflects this evolved knowledge about adolescent dysmenorrhoea.

Clinical challenges follow on from research challenges; limitations in research translate into limitations or gaps in understanding that risk manifestation in guidance, in how clinicians share information, make referral and diagnostic decisions, and counsel young people about treatment options. This extends into how we understand and define “normal” and into knowing how to educate and enable etc.

Delays in diagnosis of endometriosis are testament to the complexity of differentiating primary from secondary dysmenorrhoea: arguably clinicians and patients are ill-served if this is not recognised and acknowledged in clinical practice. It might be better to call the symptom dysmenorrhoea just that, treating and validating all dysmenorrhoea, while keeping our clinic room doors and minds open.

## Data Availability

Publicly available datasets were analyzed in this study. This data can be found here: The original dataset is the systematic review published by this journal (16).

## Footnotes

^1^https://icd.who.int/en.

## References

[1] Armour M Ferfolja T Curry C Hyman MS Parry K Chalmers KJ The prevalence and educational impact of pelvic and menstrual pain in Australia: a national online survey of 4,202 young women aged 13–25 years. J Pediatr Adolesc Gynecol. (2020) 33(5):511–8. 10.1016/j.jpag.2020.06.007 32544516

[2] Randhawa AE Tufte-Hewett AD Weckesser AM Jones GL Hewett FG. Secondary school girls’ experiences of menstruation and awareness of endometriosis: a cross-sectional study. J Pediatr Adolesc Gynecol. (2021) 34(5):643–8. 10.1016/j.jpag.2021.01.021 33548448

[3] Parker MA Sneddon AE Arbon P. The menstrual disorder of teenagers (MDOT) study: determining typical menstrual patterns and menstrual disturbance in a large population-based study of Australian teenagers. BJOG. (2010) 117(2):185–92. 10.1111/j.1471-0528.2009.02407.x 19874294

[4] Suvitie PA Hallamaa MK Matomaki JM Makinen JI Perheentupa AH. Prevalence of pain symptoms suggestive of endometriosis among Finnish adolescent girls (TEENMAPS study). J Pediatr Adolesc Gynecol. (2016) 29(2):97–103. 10.1016/j.jpag.2015.07.001 26169662

[5] Armour M Parry K Manohar N Holmes K Ferfolja T Curry C The prevalence and academic impact of dysmenorrhea in 21,573 young women: a systematic review and meta-analysis. J Womens Health. (2019) 28(8):1161–71. 10.1089/jwh.2018.7615 31170024

[6] De Sanctis V Soliman A Bernasconi S Bianchin L Bona G Bozzola M Primary dysmenorrhea in adolescents: prevalence, impact and recent knowledge. Pediatr Endocrinol Rev. (2015) 13(2):512–20. PMID: 26841639 26841639

[7] Anonymous. ACOG committee opinion no. 760: dysmenorrhea and endometriosis in the adolescent. Obstet Gynecol. (2018) 132(6):e249–58. 10.1097/AOG.0000000000002978 30461694

[8] Harel Z. Dysmenorrhea in adolescents and young adults: etiology and management. J Pediatr Adolesc Gynecol. (2006) 19(6):363–71. 10.1016/j.jpag.2006.09.001 17174824

[9] Horne AW Missmer SA. Pathophysiology, diagnosis, and management of endometriosis. Br Med J. (2022) 379:e070750. 10.1136/bmj-2022-070750 36375827

[10] Brosens I Gordts S Benagiano G. Endometriosis in adolescents is a hidden, progressive and severe disease that deserves attention, not just compassion. Hum Reprod. (2013) 28(8):2026–31. 10.1093/humrep/det243 23739215 PMC3712662

[11] Shah DK Missmer SA. Scientific investigation of endometriosis among adolescents. J Pediatr Adolesc Gynecol. (2011) 24(5):S18–9. 10.1016/j.jpag.2011.07.008 21856546

[12] Shim JY Laufer MR. Adolescent endometriosis: an update. J Pediatr Adolesc Gynecol. (2020) 33(2):112–9. 10.1016/j.jpag.2019.11.011 31812704

[13] Janssen E Rijkers A Hoppenbrouwers K. Meuleman C d'Hooghe T. Prevalence of endometriosis diagnosed by laparoscopy in adolescents with dysmenorrhea or chronic pelvic pain: a systematic review. Hum Reprod Update. (2013) 19(5):570–82. 10.1093/humupd/dmt016 23727940

[14] Dixon S Ranger TA Burchardt J Patone M Snelling AJ Vincent K Exploring the interface between adolescent dysmenorrhoea and endometriosis: a protocol for a cohort and nested case–control study within the QResearch database. BMJ Open. (2023) 13(2):e069984. 10.1136/bmjopen-2022-069984 36787972 PMC9930556

[15] Dixon S McNiven A Talbot A Hinton L. Navigating possible endometriosis in primary care: a qualitative study of GP perspectives. Br J Gen Pract. (2021) 71(710):e668–76. 10.3399/BJGP.2021.0030 33950856 PMC8340732

[16] Dixon S Hirst J Taghinejadi N Duddy C Vincent K Ziebland S. What is known about adolescent dysmenorrhoea in (and for) community health settings? Front Reprod Health. (2024) 6:1394978. 10.3389/frph.2024.1394978 39109074 PMC11300274

[17] Jutel AG. Putting a Name to it: Diagnosis in Contemporary Society. Baltimore, Maryland, USA: John Hopkins University Press (2014).

[18] Jutel A Nettleton S. Towards a sociology of diagnosis: reflections and opportunities. Soc Sci Med. (2011) 73(6):793–800. 10.1016/j.socscimed.2011.07.014 21868144

[19] Nettleton S. The Sociology of Health and Illness. 4th ed. Cambridge, UK: Polity press (2020).

[20] Strange J-M. Menstrual fictions: languages of medicine and menstruation, c. 1850–1930. Womens Hist Rev. (2000) 9(3):607–28. 10.1080/09612020000200260

[21] O'Flynn N. Menstrual symptoms: the importance of social factors in women’s experiences. Br J Gen Pract. (2006) 56(533):950–7. Erratum in: *Br J Gen Pract.* (2007) 57(535):156. PMID: . PMCID: PMC1934056 17132384 PMC1934056

[22] Heald FP Jr. Masland RP Jr. Sturgis SH Gallagher JR. Dysmenorrhea in adolescence. Pediatrics. (1957) 20(1):121–7. 10.1542/peds.20.1.121 13441385

[23] Gardner J. Adolescent menstrual characteristics as predictors of gynaecological health. Ann Hum Biol. (1983) 10(1):31–40. 10.1080/03014468300006161 6838154

[24] Lawlor CL Davis AM. Primary dysmenorrhea. Relationship to personality and attitudes in adolescent females. J Adolesc Health Care. (1981) 1(3):208–12. 10.1016/S0197-0070(81)80058-7 7333923

[25] Holmlund U. The experience of dysmenorrhea and its relationship to personality variables. Acta Psychiatr Scand. (1990) 82(2):182–7. 10.1111/j.1600-0447.1990.tb01379.x 2239364

[26] Frisk M Widholm O Hortling H. Dysmenorrhœa—Psyche and Soma in Teenagers. Acta Obstet Gynecol Scand. (1965) 44(2):339–47. 10.3109/00016346509155870 5834182

[27] Batt RE Sturgis SH. Adolescents’ gynecologic problems. Medical aspects. Clin Pediatr (Phila). (1968) 7(1):17–23. 10.1177/000992286800700108 5635212

[28] Sloan D. Pelvic pain and dysmenorrhea. Pediatr Clin North Am.. (1972) 19(3):669–80. 10.1016/s0031-3955(16)32745-6 5064693

[29] Schauffler GC. Dysmenorrhea in and near puberty. Ann N Y Acad Sci. (1967) 142(3):794–800. 10.1111/j.1749-6632.1967.tb14692.x 5231262

[30] Tindall VR. Dysmenorrhoea. Br Med J. (1971) 1(5744):329–31. 10.1136/bmj.1.5744.329 5100266 PMC1794860

[31] Harel Z. Dysmenorrhea in adolescents and young adults: from pathophysiology to pharmacological treatments and management strategies. Expert Opin Pharmacother. (2008) 9(15):2661–72. 10.1517/14656566.9.15.2661 18803452

[32] Harel Z Lilly C Riggs S Vaz R Drazen J. Urinary leukotriene (LT) E(4) in adolescents with dysmenorrhea: a pilot study. J Adolesc Health. (2000) 27(3):151–4. 10.1016/S1054-139X(00)00123-3 10960212

[33] Alvin PE Litt IF. Current status of the etiology and management of dysmenorrhea in adolescence. Pediatrics. (1982) 70(4):516–25. 10.1542/peds.70.4.516 6812011

[34] Khoiny FE. Adolescent dysmenorrhea. J Pediatr Health Care. (1988) 2(1):29–37. 10.1016/0891-5245(88)90057-0 3339527

[35] Stromberg P Akerlund M Forsling ML Granstrom E Kindahl H. Vasopressin and prostaglandins in premenstrual pain and primary dysmenorrhea. Acta Obstet Gynecol Scand. (1984) 63(6):533–8. 10.3109/00016348409156715 6542295

[36] Jay MS DuRant RH. The patient with dysmenorrhea. Postgrad Med. (1983) 73(4):103–6, 9–11. 10.1080/00325481.1983.11698351 6682221

[37] Comerci GD. Symptoms associated with menstruation. Pediatr Clin N Am. (1982) 29(1):177–200. 10.1016/S0031-3955(16)34116-5 7058070

[38] Zola IK. Medicine as an institution of social control. Sociol Rev. (1972) 20(4):487–504. 10.1111/j.1467-954X.1972.tb00220.x 4645802

[39] Available online at: https://www.who.int/standards/classifications/classification-of-diseases (Accessed July 11, 2024).

[40] Available online at: https://icd.who.int/ct11/icd11_mms/en/release (Accessed July 11, 2024).

[41] Available online at: https://cks.nice.org.uk/topics/dysmenorrhoea/ (Accessed July 11, 2024).

[43] Kho KA Shields JK. Diagnosis and management of primary dysmenorrhea. JAMA. (2020) 323(3):268–9. 10.1001/jama.2019.16921 31855238

[42] Durain D. Primary dysmenorrhea: assessment and management update. J Midwifery Womens Health. (2004) 49(6):520–8. 10.1016/j.jmwh.2004.08.013 15544981

[44] Harel Z. Dysmenorrhea in adolescents and young adults: an update on pharmacological treatments and management strategies. Expert Opin Pharmacother. (2012) 13(15):2157–70. 10.1517/14656566.2012.725045 22984937

[45] Harel Z. A contemporary approach to dysmenorrhea in adolescents. Paediatr Drugs. (2002) 4(12):797–805. 10.2165/00128072-200204120-00004 12431132

[46] Sachedina A Todd N. Dysmenorrhea, endometriosis and chronic pelvic pain in adolescents. J Clin Res Pediatr Endocrinol. (2020) 12:7–17. 10.4274/jcrpe.galenos.2019.2019.S0217 32041388 PMC7053437

[47] Harada T. Dysmenorrhea and endometriosis in young women. Yonago Acta Med. (2013) 56(4):81–4. PMID: 24574576 24574576 PMC3935015

[48] Chauhan M Kala J. Relation between dysmenorrhea and body mass index in adolescents with rural versus urban variation. J Obstetr Gynaecol India. (2012) 62(4):442–5. 10.1007/s13224-012-0171-7 PMC3500946 23904707

[49] Monday I Anthony P Olunu E Otohinoyi D Abiodun S Owolabi A Prevalence and correlation between diet and dysmenorrhea among high school and college students in Saint Vincent and grenadines. Open Access Maced J Med Sci. (2019) 7(6):920–4. 10.3889/oamjms.2019.205 30976334 PMC6454168

[50] Sachedina A Abu Bakar M Dunford AM Morris A Nur Azurah AG Grover SR. Dysmenorrhea in young people: experiences from a tertiary center with a focus on conservative management. J Obstetr Gynaecol Res. (2021) 47(1):352–8. 10.1111/jog.14532 33084069

[51] Wu M-H Shoji Y Chuang P-C Tsai S-J. Endometriosis: disease pathophysiology and the role of prostaglandins. Expert Rev Mol Med. (2007) 9(2):1–20. 10.1017/S146239940700021X 17227592

[52] Sacco K Portelli M Pollacco J Schembri-Wismayer P Calleja-Agius J. The role of prostaglandin E2 in endometriosis. Gynecol Endocrinol. (2012) 28(2):134–8. 10.3109/09513590.2011.588753 22003899

[53] Kuznetsov L Dworzynski K Davies M Overton C, Guideline Committee. Diagnosis and management of endometriosis: summary of NICE guidance. *BMJ*. (2017) 358:j3935. 10.1136/bmj.j3935. Erratum in: *BMJ*. (2017) 358:j4227. 10.1136/bmj.j3935 28877898

[54] Becker CM Bokor A Heikinheimo O Horne A Jansen F Kiesel L ESHRE Guideline: endometriosis. Hum Reprod Open. (2022) 2022(2):hoac009. 10.1093/hropen/hoac009 35350465 PMC8951218

[55] de Sanctis V Matalliotakis M Soliman AT Elsefdy H Di Maio S Fiscina B. A focus on the distinctions and current evidence of endometriosis in adolescents. Best Pract Res Clin Obstet Gynaecol. (2018) 51:138–50. 10.1016/j.bpobgyn.2018.01.023 29548642

[56] Brown J Crawford TJ Allen C Hopewell S Prentice A. Nonsteroidal anti-inflammatory drugs for pain in women with endometriosis. Cochrane Database Syst Rev. (2017) 1(1):CD004753. 10.1002/14651858.CD004753.pub4 28114727 PMC6464974

[57] Hewitt G. Dysmenorrhea and endometriosis: diagnosis and management in adolescents. Clin Obstetr Gynecol. (2020) 63(3):536–43. 10.1097/GRF.0000000000000540 32366763

[58] Hoppenbrouwers K Roelants M Meuleman C Rijkers A Van Leeuwen K Desoete A Characteristics of the menstrual cycle in 13-year-old Flemish girls and the impact of menstrual symptoms on social life. Eur J Pediatr. (2016) 175(5):623–30. 10.1007/s00431-015-2681-7 26670027

[59] Sultan C Jeandel C Paris F Trimeche S. Adolescent dysmenorrhea. Endocr Dev. (2004) 7:140–7. 10.1159/000077082 15052996

[60] Jeon GE Cha NH Sok SR. Factors influencing the dysmenorrhea among Korean adolescents in middle school. J Phys Ther Sci. (2014) 26(9):1337–43. 10.1589/jpts.26.1337 25276012 PMC4175233

[61] Flug D Largo RH Prader A. Symptoms related to menstruation in adolescent Swiss girls: a longitudinal study. Ann Hum Biol. (1985) 12(2):161–8. 10.1080/03014468500007651 4039116

[62] El-Gilany AH Badawi K El-Fedawy S. Epidemiology of dysmenorrhoea among adolescent students in Mansoura, Egypt. East Mediterr Health J. (2005) 11(1–2):155–63. PMID: 16532684 16532684

[63] Abu Helwa HA Mitaeb AA Al-Hamshri S Sweileh WM. Prevalence of dysmenorrhea and predictors of its pain intensity among Palestinian female university students. BMC Womens Health. (2018) 18(1):18. 10.1186/s12905-018-0516-1 29334974 PMC5769430

[64] DiVasta AD Vitonis AF Laufer MR Missmer SA. Spectrum of symptoms in women diagnosed with endometriosis during adolescence vs adulthood. Am J Obstetr Gynecol. (2018) 218(3):324.e1–11. 10.1016/j.ajog.2017.12.007 29247637

[65] Gunn HM Tsai MC McRae A Steinbeck KS. Menstrual patterns in the first gynecological year: a systematic review. J Pediatr Adolesc Gynecol. (2018) 31(6):557–65.e6. 10.1016/j.jpag.2018.07.009 30064002

[66] Akman AO Bozdag G Kizilkan MP Akgul S Derman O Kanbur N. Menstrual cycle pain is independent of ovulation in adolescents with primary dysmenorrhea. J Pediatr Adolesc Gynecol. (2021) 34(5):635–42. 10.1016/j.jpag.2021.04.001 33910090

[67] Saridogan E. Endometriosis in teenagers. Womens Health. (2015) 11(5):705–9. 10.2217/whe.15.58 26315257

[68] Hirsch M Dhillon-Smith R Cutner AS Yap M Creighton SM. The prevalence of endometriosis in adolescents with pelvic pain: a systematic review. J Pediatr Adolesc Gynecol. (2020) 33(6):623–30. 10.1016/j.jpag.2020.07.011 32736134

[69] Knox B Ong YC Bakar MA Grover SR. A longitudinal study of adolescent dysmenorrhoea into adulthood. Eur J Pediatr. (2019) 178(9):1325–32. 10.1007/s00431-019-03419-3 31292729

[70] Ragab A Shams M Badawy A Alsammani MA. Prevalence of endometriosis among adolescent school girls with severe dysmenorrhea: a cross sectional prospective study. Int J Health Sci. (2015) 9(3):273–81. 10.12816/0024694 PMC4633191 26609292

[71] Vercellini P Eskenazi B Consonni D Somigliana E Parazzini F Abbiati A Oral contraceptives and risk of endometriosis: a systematic review and meta-analysis. Hum Reprod Update. (2011) 17(2):159–70. 10.1093/humupd/dmq042 20833638

[72] Chapron C Borghese B Streuli I de Ziegler D. Markers of adult endometriosis detectable in adolescence. J Pediatr Adolesc Gynecol. (2011) 24(5):S7–12. 10.1016/j.jpag.2011.07.006 21856548

[73] Cramer DW Missmer SA. The epidemiology of endometriosis. Ann N Y Acad Sci. (2002) 955:11–22; discussion 34–6, 396–406. 10.1111/j.1749-6632.2002.tb02761.x 11949940

[74] Clemenza S Vannuccini S Capezzuoli T Meleca CI Pampaloni F Petraglia F. Is primary dysmenorrhea a precursor of future endometriosis development? Gynecol Endocrinol. (2021) 37(4):287–93. 10.1080/09513590.2021.1878134 33569996

[75] Motashaw ND. Endometriosis in young girls. Contrib Gynecol Obstetr. (1987) 16:22–7. 10.1159/000414843 2961505

[76] Zannoni L Giorgi M Spagnolo E Montanari G Villa G Seracchioli R. Dysmenorrhea, absenteeism from school, and symptoms suspicious for endometriosis in adolescents. J Pediatr Adolesc Gynecol. (2014) 27(5):258–65. 10.1016/j.jpag.2013.11.008 24746919

[77] Farquhar CM Roberts H Okonkwo QL Stewart AW. A pilot survey of the impact of menstrual cycles on adolescent health. Aust N Z J Obstet Gynaecol. (2009) 49(5):531–6. 10.1111/j.1479-828X.2009.01062.x 19780739

[78] Sahin ME Sahin E Madendag Y Madendag IC Tayyar AT Ozdemir F The effect of anterior uterocervical angle on primary dysmenorrhea and disease severity. Pain Res Manag. (2018) 2018:9819402. 10.1155/2018/9819402 30305856 PMC6166362

[79] Bahrami A Bahrami-Taghanaki H Khorasanchi Z Timar A Jaberi N Azaryan E Menstrual problems in adolescence: relationship to serum vitamins A and E, and systemic inflammation. Arch Gynecol Obstet. (2020) 301(1):189–97. 10.1007/s00404-019-05343-1 31734759

[80] Bush D Brick E East MC Johnson N. Endometriosis education in schools: a New Zealand model examining the impact of an education program in schools on early recognition of symptoms suggesting endometriosis. Aust N Z J Obstet Gynaecol. (2017) 57(4):452–7. 10.1111/ajo.12614 28349513

[81] Angelhoff C Grundström H. Supporting girls with painful menstruation-a qualitative study with school nurses in Sweden. J Pediatr Nurs. (2023) 68:e109–15. 10.1016/j.pedn.2022.11.022 36446692

[82] Martin E. The Woman in the Body: A Cultural Analysis of Reproduction. Boston, Massachusetts, USA: Beacon Press (2001).

[83] Elinor Crichton unwell women.

[84] Seear K. The etiquette of endometriosis: stigmatisation, menstrual concealment and the diagnostic delay. Soc Sci Med. (2009) 69(8):1220–7. 10.1016/j.socscimed.2009.07.023 19699572

[85] Armour M Parry K Al-Dabbas MA Curry C Holmes K MacMillan F Self-care strategies and sources of knowledge on menstruation in 12,526 young women with dysmenorrhea: a systematic review and meta-analysis. PLoS One. (2019) 14(7):e0220103. 10.1371/journal.pone.0220103 31339951 PMC6655766

[86] Smith CA Armour M Zhu X Li X Lu ZY Song J. Acupuncture for dysmenorrhoea. Cochrane Database Syst Rev. (2016) (4):CD007854. 10.1002/14651858.CD007854.pub3 27087494 PMC8406933

[87] Marjoribanks J Ayeleke RO Farquhar C Proctor M. Nonsteroidal anti-inflammatory drugs for dysmenorrhoea. Cochrane Database Syst Rev. (2015) (7): CD001751. 10.1002/14651858.CD001751.pub3 26224322 PMC6953236

